# A Case Report on Invasive Mucormycosis Involving the Maxillary Region, Brain, and Chest

**DOI:** 10.7759/cureus.68873

**Published:** 2024-09-07

**Authors:** Riya Yadav, Pratapsingh Parihar, Suyash Yadav, Shweta Yadav

**Affiliations:** 1 Department of Radiodiagnosis, Jawaharlal Nehru Medical College, Acharya Vinoba Bhave Rural Hospital, Datta Meghe Institute of Higher Education and Research, Wardha, IND; 2 Department of Pathology, Shri Shankaracharya Institute of Medical Sciences, Durg, IND; 3 Department of Internal Medicine, Shri Shankaracharya Institute of Medical Sciences, Durg, IND

**Keywords:** diabetes mellitus, immunocompromised, maxilla, mucorales, mucormycosis, rhinocerebral

## Abstract

The maxilla is a facial bone with a dense blood supply. Although rare, infections, trauma, and certain metabolic disorders can lead to necrosis of the maxillary bone. The maxilla, a vital bone forming the roof of the oral cavity, is particularly susceptible to mucormycosis, a prevalent fungal infection, especially in individuals with diabetes and compromised immune systems. Here, we discuss mucormycosis-associated maxillary necrosis in a patient with uncontrolled diabetes. Early identification and treatment of this lethal fungal infection can significantly reduce its mortality and morbidity rates. Due to the maxilla's high vascularity, necrosis is uncommon. However, various infections, including bacterial, viral, and fungal, can lead to maxillary necrosis. Other potential causes include trauma, radiation, prolonged corticosteroid use, and lipid metabolism disorders (like Gaucher disease). Early diagnosis and timely treatment are crucial to significantly reducing the mortality and morbidity associated with this life-threatening fungal infection.

## Introduction

Mucormycosis encompasses various conditions caused by fungi belonging to the Mucorales order [1]. Patients typically have underlying conditions such as uncontrolled diabetes mellitus and hematological malignancies such as leukemia, or are undergoing immunosuppressive treatment [2,3]. Fungal spores, when inhaled, can initiate an infection in the nasal and paranasal sinuses. This infection can spread to orbital and cerebral tissues via blood vessels or direct invasion. The fungus invades the arteries, leading to thrombosis, which, in turn, causes necrosis of both soft and hard tissues [4]. This infection typically presents acutely and can manifest in various forms, including rhinocerebral, pulmonary, gastrointestinal, cutaneous, or widespread infection [5]. In the head and neck region, it most frequently presents as maxillary and orbital cellulitis [6].

Regarding its prevalence, mucormycosis ranks as the third most common angioinvasive fungal infection, following aspergillosis and candidiasis [7]. A computed tomography (CT) scan is useful for evaluating paranasal sinuses and nearby structures such as the eyes and brain. On a CT scan, typical findings may include soft-tissue swelling of the orbital cavity mucosa, mucoperiosteal thickening in the sinuses, bone erosion, and orbital invasion. For patients who are immunocompromised and exhibit respiratory symptoms, a chest CT scan is advised to assess for potential pulmonary mucormycosis, which can mimic pneumonia and complicate diagnosis. Imaging results in the chest might show pleural effusion, small masses, areas of consolidation, and hazy infiltrates, while bronchoalveolar lavage could detect wide, nonseptate fungal hyphae [8,9].

To improve survival outcomes in mucormycosis, it is crucial to maintain a high level of clinical suspicion, promptly identify predisposing factors, and quickly assess clinical and radiological findings. This case contributes to the growing body of knowledge on mucormycosis by underscoring its clinical manifestations and the extent of its impact on the maxilla, brain, and lungs.

## Case presentation

A 52-year-old patient, who was previously in good health, began experiencing symptoms 10 days ago, starting with a dull, aching, and intermittent pain localized to the left jaw. The pain intensified during chewing but was relieved with analgesic medication. Additionally, he developed a cough, chest pain, and shortness of breath, which were also alleviated with medication. He reported episodes of nosebleeds from the left nostril associated with coughing and a teary left eye. The patient has a known history of diabetes mellitus and has been chewing tobacco two to three times daily for the past 10 years. There is no history of trauma or COVID-19.

**Figure 1:**
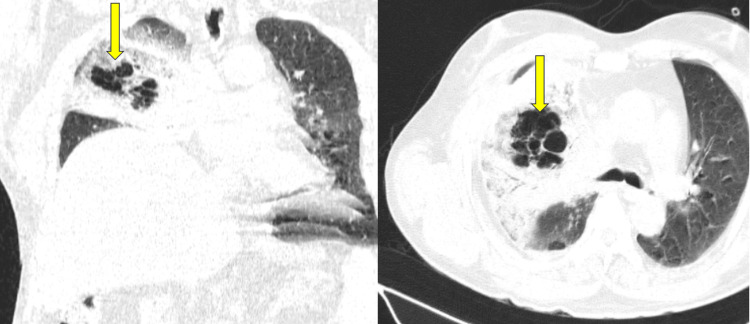
HRCT coronal and axial sections of the thorax. The yellow arrows depict the conglomerated bubbly cystic lucencies seen in the right upper lobe (bird nest appearance) HRCT: high-resolution computed tomography

The diagnostic evaluation included a high-resolution CT of the thorax, which revealed conglomerated bubbly cystic lucencies in the right upper lobe, resembling a bird's nest appearance, as illustrated in Figure 1. Additionally, a few small, variably sized preparatracheal mediastinal lymph nodes were observed, as shown in Figure 1, with some subtle areas of hyperdense soft calcification. The largest lymph node measured approximately 16 x 15 mm. Furthermore, there was significant ground-glass attenuation and consolidation around the periphery, with interlobular septal thickening affecting almost the entire right upper lobe and the right middle lobe. A minimally thin strip of multifocal right-sided pleural effusion with thickening of the interlobular septa was also noted.

Table 1 provides detailed information on the involvement of various sinuses and bones with regard to mucosal thickening and bone involvement.

**Table 1:** Involvement of sinuses and bones

Sinuses	Mucosal thickening		Bone involvement	
	Right	Left	Right	Left
			Sinus walls	Sinus walls
Maxillary sinuses	Absent	Present	Absent	Bony sclerosis along the basal and medial walls
Osteomeatal complex	Absent	Obstructed	Absent	Bony sclerosis
Frontal sinus and recess	Absent	Present	Absent	Absent
Ethmoid air cells	Absent	Present	Absent	Absent
Sphenoid sinus	Absent	Present	Absent	Absent
Turbinates	Absent	Present	Absent	Destruction of middle and inferior turbinates

**Figure 2:**
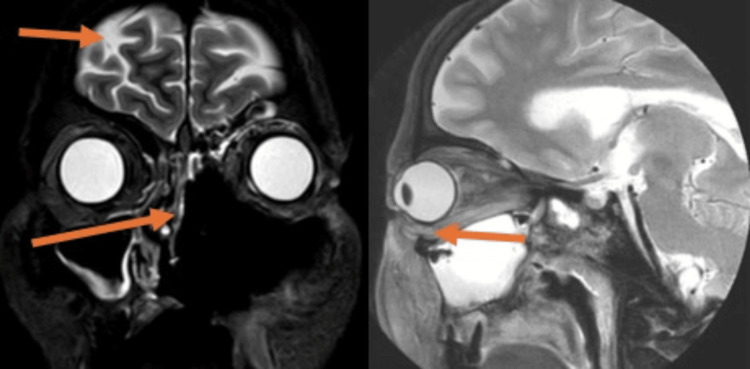
MRI coronal section STIR sequence and sagittal T2W FS sequence. The orange arrows depict meningeal involvement, and maxillary sinus with middle and inferior turbinate extension along with the protruded left globe and a bulky extraocular muscle STIR: short tau inversion recovery; T2WI: T2-weighted image; T2W FS: T2-weighted fat saturation

The MRI of the orbit reveals a "black turbinate sign," as illustrated in Figure 2, accompanied by involvement of the left maxillary sinus and a mildly deviated nasal septum toward the right. There are patchy areas of smooth meningeal enhancement in the left basifrontal and temporal regions, which are indicative of focal meningitis. Swelling and increased contrast are observed in the left side of periantral fat pads, pterygopalatine fossa, infratemporal fossa, masticator space, buccal space, and parapharyngeal space. Furthermore, minor erosions are seen on the left side of the hard palate. The left globe is protruded, suggesting proptosis, with the left extraocular muscles appearing bulky, showing signs of edema and postcontrast enhancement, as seen in Figure 2. Inflammation is observed in the left extraocular and intraocular fat, which is consistent with orbital cellulitis. Perineural enhancement is noted in the left orbital portion of the optic nerve, suggesting optic perineuritis. An area of altered signal intensity in the left temporal lobe shows hypoenhancement, which is indicative of encephalitis. Furthermore, vessel wall enhancement is identified in the left lacerum segment of the internal carotid artery, suggesting vasculitis.

**Figure 3:**
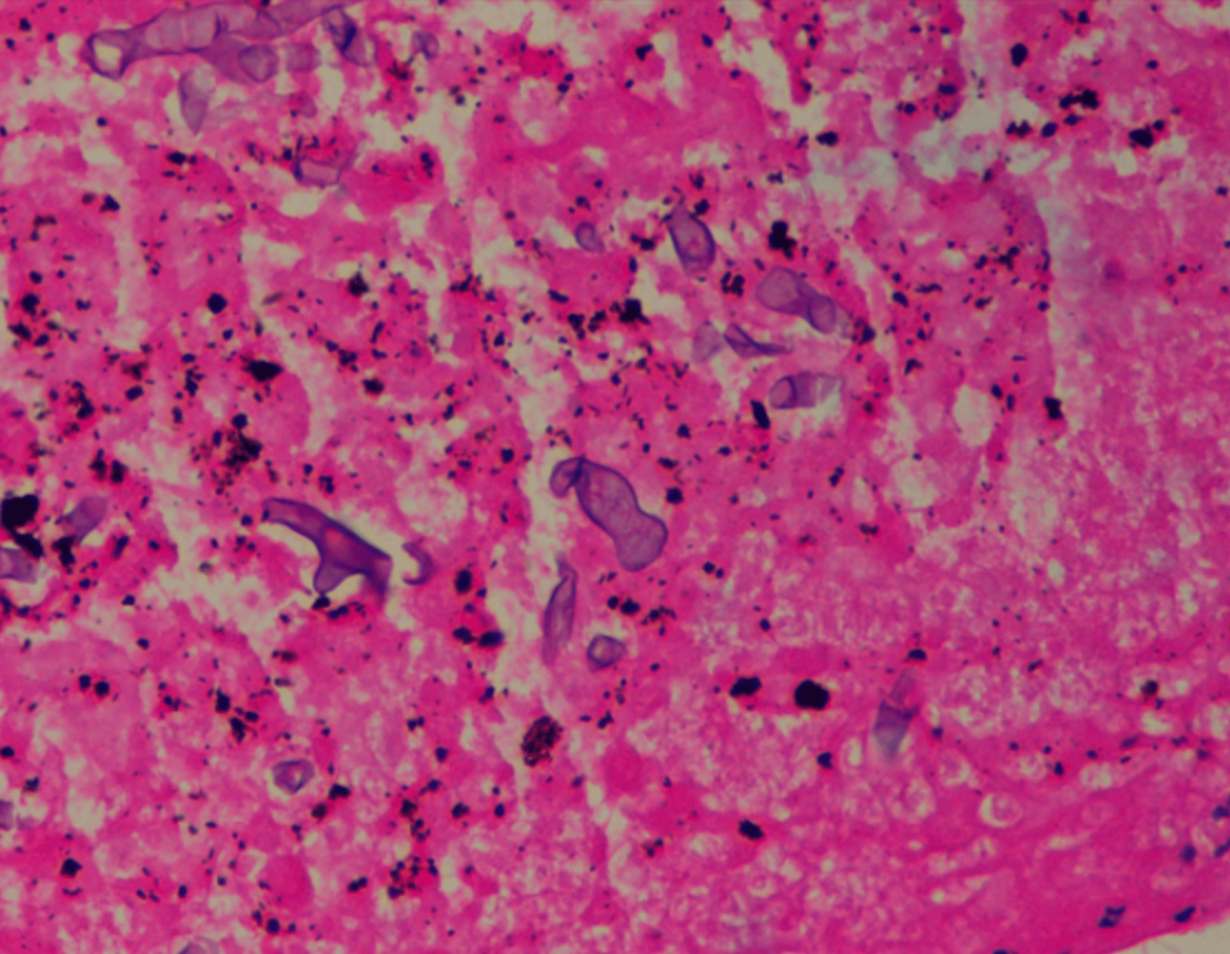
H&E staining from the lung biopsy taken, showing pauciseptate fungal hyphae with acute angle branching, consistent with Mucorales H&E: hematoxylin and eosin

The lung biopsy's hematoxylin and eosin staining is consistent with Mucorales's findings, as depicted in Figure 3*.*

It is highly advised that therapy be initiated immediately in any immunocompromised patient suspected of having mucormycosis. While obtaining a diagnosis is crucial, it should not delay the start of treatment. In this patient's case, treatment began with liposomal amphotericin B at 10 mg/kg per day. Despite this, the patient's condition rapidly deteriorated, going in for sepsis with respiratory acidosis, and he ultimately passed away from acute cardiorespiratory failure.

## Discussion

Mucorales fungi, commonly found in soil and decomposing organic material, are thermotolerant but usually do not cause disease due to their low virulence. The rise in mucormycosis cases is linked to the increasing prevalence of immunosuppressive conditions, improved survival rates among cancer and transplant patients, and the wider use of immunosuppressive drugs for autoimmune disorders. The primary mode of infection is through inhalation of spores that can settle in the paranasal sinuses and lungs. Less frequently, mucormycosis can occur through ingestion or direct skin inoculation. Risk factors include diabetes mellitus, malnutrition, certain types of cancer, renal failure, organ transplants, burns, immunosuppressive therapy, cirrhosis, and AIDS. Patients with diabetic ketoacidosis and those on dialysis receiving the iron chelator deferoxamine are particularly vulnerable. Mucormycosis can impact various body systems, with potential infection sites including the lungs, central nervous system, paranasal sinuses, gastrointestinal tract, and skin. The clinical symptoms are diverse but tend to progress rapidly. Symptoms largely depend on the fungal entry route and the patient's underlying health conditions [10,11].

In this case, the patient exhibited symptoms such as nosebleeds and had a known history of diabetes, both of which contribute to the disease's pathophysiology. Rhinocerebral mucormycosis begins when spores are inhaled into the paranasal sinuses. These spores infiltrate the surrounding tissues and infect blood vessels. The infection initially manifests as nasal congestion or discharge but can rapidly progress to symptoms such as facial numbness, blurred vision, nasofrontal headache, eye pain, fever, double vision, and swelling around the eyes. Typical intranasal lesions are painless ulcers with exudate and necrotic tissue, usually progressing swiftly within a few days. In immunocompromised patients who exhibit persistent nasal symptoms, performing a biopsy to rule out mucormycosis, also known as invasive fungal sinusitis, is crucial. Diagnosing mucormycosis depends heavily on the availability of imaging techniques, skilled personnel, and thorough mycological and histological investigations. Patients suspected of having mucormycosis should be immediately referred to a facility equipped to provide the highest level of care. Routine blood tests are seldom diagnostic but can help identify neutropenia as a related risk factor. Imaging is essential in evaluating the extent of the disease. When rhinocerebral mucormycosis is suspected, the first step involves an endoscopic evaluation and sinus biopsy to assess for tissue necrosis and collect samples. The identification of characteristic hyphae can lead to a presumptive diagnosis. The middle turbinate is the most reliable biopsy site, offering the highest yield and accuracy. A CT scan helps assess adjacent structures, such as the eyes and brain, with typical findings including soft-tissue swelling of the cavity mucosa, sinus mucoperiosteal thickening, bone erosion, and orbital invasion. In immunosuppressed patients who exhibit respiratory symptoms, a chest CT scan is recommended to evaluate for potential pulmonary mucormycosis. This infection often mimics pneumonia, complicating diagnosis. Chest radiologic findings may include pleural effusion, nodules, consolidation, and ground-glass infiltrates. Bronchoalveolar lavage can reveal broad, nonseptate hyphae [12-15]. To confirm the infection, nonpigmented hyphae indicating tissue invasion must be identified in tissue sections stained with hematoxylin-eosin [16].

The most significant prognostic factor is the ability to restore normal immune function. If this cannot be achieved, the prognosis is generally poor. However, the prognosis improves if immunocompetence can be restored, even temporarily [16]. Unfortunately, in our case, the prognosis was grim, as the patient ultimately succumbed to respiratory failure.

Liposomal amphotericin B has been effective in treating mucormycosis involving multiple organs. Daily doses have ranged from 1 to 10 mg/kg, with higher doses generally yielding better response rates [15].

Complications of mucormycosis can be divided into those resulting from the disease itself and those caused by antifungal treatment. Disease-related complications include cavernous sinus thrombosis, disseminated infection, periorbital destruction, palatine ulcers, osteomyelitis, and death. Treatment-related complications include nephrotoxicity, hypokalemia, and prolonged hospitalization, particularly with the use of deoxycholate amphotericin B [17]. Our case illustrates the rapid progression of mucormycosis, highlighting various diagnostic findings. This case provides valuable insights into the presentations observed in CT, MRI, and biopsy findings specific to our region. We identified several classic features that we wish to share with fellow diagnosticians to contribute to a better understanding of the disease onset, pathophysiology, investigative workups, and potential treatments, along with associated complications.

## Conclusions

While invasive mycotic infections can sometimes resemble cancer in certain imaging aspects, these infections should be diagnosed within the appropriate clinical context. Early identification and management of mucormycosis is crucial to prevent potentially fatal outcomes and to ensure comprehensive, patient-centered care. Our patient exhibited several significant findings, including orbital cellulitis with myositis, optic perineuritis, localized meningitis, encephalitis, vasculitis, and a bird nest appearance in the right upper lung lobe, as well as proptosis. For a definitive diagnosis, immunohistochemistry and histopathological testing are recommended. Providing comprehensive care for these patients requires a collaborative effort among healthcare practitioners. By working together, medical professionals can ensure timely and accurate diagnosis and effective management of mucormycosis, thus improving patient outcomes.
